# Suitability of saliva for Tuberculosis diagnosis: comparing with serum

**DOI:** 10.1186/s12879-017-2687-z

**Published:** 2017-08-31

**Authors:** Anna Ritah Namuganga, Novel N. Chegou, Paul Mubiri, Gerhard Walzl, Harriet Mayanja-Kizza

**Affiliations:** 1 Joint Clinical Research Centre, Kampala, Uganda; 2 Division of Molecular Biology and Human Genetics, Department of Biomedical Sciences, DST/NRF Centre of Excellence for Biomedical Tuberculosis Research and SAMRC Centre for Tuberculosis Research, Faculty of Medicine and Health Sciences, Stellenbosch University, Cape Town, South Africa; 3 Uganda – Case Western Research Collaboration, Mulago-Kampala, Uganda; 4 College of Health Sciences, Makerere University, Kampala, Uganda

**Keywords:** Biomarkers, Quantiferon, Saliva

## Abstract

**Background:**

In the search for fast, simple and better ways for diagnosis of tuberculosis (TB), there is need to discover and evaluate new biomarkers that are found in samples other than sputum to determine their effectiveness. This study examined the utility of saliva vis-a-vis serum by evaluating levels of biomarkers found in saliva and serum from TB suspects.

**Methods:**

Study enrolled tuberculosis suspects. Sputum MGIT was used as the gold standard for active TB. Quantiferon gold-In tube assay was done to identify exposure to *Mycobacterium tuberculosis* (*M.tb*). Multiplex assay was run for 10 markers using a 10 plex customized kit from Bio-Rad Laboratories.

**Results:**

There was a significant difference between saliva and serum marker levels. Saliva had significantly higher levels of GM-CSF and VEGF. Serum had higher levels of MIP-1a, b, TNF-a, G-CSF and IFN-g. Serum levels of IL-6, VEGF and TNF-a were significantly different between participants with active TB disease and those with other respiratory diseases.

**Conclusion:**

Salivary TB biomarkers are worth the search to evaluate their ability to differentiate between TB disease states for generation of a non invasive point of care test for TB diagnosis.

## Background

Globally, tuberculosis (TB) ranks alongside HIV as the leading cause of mortality and morbidity [1]. The diagnosis of the disease remains a major problem, due to several shortcomings in the currently available diagnostic tests. Smear microscopy, the most widely available TB diagnostic test has poor sensitivity [2]. Sputum culture, the gold standard has a long turnaround time (up to 42 days) [3], whereas the recently developed GeneXpert MTB/RIF test (Cepheid Inc., Sunnyvale, USA), although rapid, is expensive, amongst other limitations which hamper its use in resource-poor countries [4]. All these tests also depend on the quality of sputum provided by the patient, to yield reliable results. This is highly problematic in children who cannot cough, and in individuals with extra pulmonary TB.

Current research has given birth to immunodiagnostic tests including the interferon gamma release assays (IGRA) such as the Quantiferon TB Gold and the T-SPOT assays. These tests require a blood draw to evaluate the body’s T cell response towards *Mycobacterium tuberculosis* (*Mtb),* as a means to diagnose infection with *Mtb* infection. These assays are based on the principle that individuals who have previously been exposed to *Mtb* habour pre-activated T cells in circulation, which rapidly respond with the secretion of IFN-γ after re-challenge in vitro, with *Mtb* specific antigens. IGRAs have been shown to be useful in the diagnosis of *Mtb* infection especially in comparison with the tuberculin skin test. However, they are not able to discriminate with latent *Mtb* infection and active TB disease. This therefore means that the assays are not useful in settings with high prevalence of latent TB. Another limitation of these assays is the fact that they are overnight assays, requiring a second visit to the hospital. Assays based on ex-vivo samples such as serum, saliva or whole blood, may be more beneficial as they may lead to a more rapid diagnosis, and tests based on such assays may be easily converted to point-of-care tests.

When compared to serum, saliva has advantages which include; low protein content, easy non invasive collection and ease of storage [5–7]. Saliva has previously been used for molecular DNA testing in diagnosis of systemic diseases like hepatitis [8] HIV, renal diseases, cardiovascular diseases, autoimmune diseases, cancer, diabetes and other infectious diseases [9]. Recently, more studies have ventured into the search for biomarkers of TB in saliva [10, 11]. Compared to blood, saliva has advantages as a specimen for TB diagnosis which include none-invasiveness, no need for skilled personnel for collection, none clotting ability and ease to handle [12]. A study by Phalane et al. [13], compared serum with saliva and it was shown that some host inflammatory biomarkers are expressed in much higher concentrations in saliva than are in blood. Further studies also showed that some of the host markers detected in saliva showed potential as diagnostic biomarkers for TB disease [10, 11]. However all these previous TB studies have only been done on samples collected from a single study site. It is known from previous immunological studies [14] that immune responses tend to differ in patients recruited from different African countries, thereby highlighting the need for potential immunological based biomarkers to be investigated in different geographical regions. In the present study, we evaluated the expression of host biomarkers in serum in comparison to saliva, and further investigated whether any of these biomarkers had potential in differentiating active TB disease from latent or no TB infection, in individuals with presumed TB disease, recruited from Mulago hospital study site in Uganda. Replication of the findings from previous South African studies [10, 11, 13] in the present study would make the case for further investigation of the candidate markers identified so far and other recently identified markers in future larger studies and ultimately, the possible development of fiend-friendly TB diagnostic tests based on such salivary signatures. Furthermore, as saliva is a mucosal/airway linked sample and is relatively closer to the site of TB disease than peripheral blood, saliva may be a more informative sample for biomarker discovery purposes.

## Methods

### Study participants

Participants enrolled in this study were part of a bigger African European Tuberculosis consortium (AETBC) study that started in November 2010 and ended in December 2012. This study enrolled adults with signs and symptoms suggestive of TB disease (TB suspects), prior to the establishment of a clinical diagnosis. Ugandan study participants were recruited from within 25 km of Mulago Hospital in Kampala. All study participants had had cough for at least 2 weeks, in addition to any other TB symptoms including fever, night sweats, unintentional weight loss, chest pain, haemoptysis and contact with an active TB case. These individuals also had no history of TB treatment in the preceding 3 months. Those who gave informed consent to participate in the study were enrolled, samples collected and stored at -80 °C. Of all the PTB suspects enrolled in the bigger study, only 78 participants were selected. Serum and saliva samples from these 78 PTB suspects were used for this study. The study received ethical approval from the Uganda National Council of Science and Technology (UNCST), Makerere University College of Health Sciences (MU-CHS) as well as the Joint Clinical Research Centre (JCRC) institutional review boards.

All participants had chest x-ray, smear and culture done for diagnosis of active pulmonary TB (PTB). Sputum samples from all study participants were cultured using the MGIT method (BD Biosciences) and positive cultures were speciated to confirm *Mtb* complex, regardless of the smear result.

### Classification of study participants and reference standard

Using a combination of clinical, radiological, and laboratory findings, participants were classified as definite PTB cases, probable TB cases, participants with other respiratory diseases (ORD). A positive culture result was used to classify study participants as active PTB disease and a negative culture accompanied by clinical features, radiological findings was used to classify those with other respiratory diseases (ORD) as described in the recent paper by Chegou et al. [15]. Briefly, ORD cases had a range of other diagnoses, including upper and lower respiratory tract infections (viral and bacterial infections, although attempts to identify organisms by bacterial or viral cultures were not made), and acute exacerbations of chronic obstructive pulmonary disease or asthma. In assessing the accuracy of host biosignatures in the diagnosis of TB disease, all the definite and probable TB cases were classified as active PTB, and then compared to the ORD cases. In addition, those with negative culture (ORD) were re classified using the Quantiferon Gold In Tube assay. The latent TB infection group had positive quantiferon results while the No TB infection group had a negative quantiferon result.

### Sample collection and processing

Blood for serum separation was drawn into 8 ml serum separation tubes (BD Biosciences) and transported to the laboratory at ambient temperature. This was centrifuged at 2000×g for 10 min at room temperature. Serum was then harvested, aliquoted and stored at -80 °C until use. Saliva was drawn in salivette tubes (Sarsedt, Germany) according to manufacturer’s instruction and transported to the lab on ice. These were centrifuged at 1000×g for 2 min, aliquoted into labeled tubes and stored at -80 °C until use.

Sputum samples were collected from all participants and cultured using MGIT method (BD Biosciences). All samples that demonstrated growth of microorganisms were examined for acid-fast bacilli using the the Ziehl–Neelsen method followed by either Capilia TB testing (TAUNS, Numazu, Japan) or standard molecular methods, to confirm the isolation of organisms of the *M.tb* complex. Quantiferon Gold In Tube assay was used to identify exposure to *Mycobacterium tuberculosis.*

### Immunological assays

The Quantiferon Gold In Tube assay (QFT) was performed on all study participants in order to diagnose *Mtb* infection especially in the patients with ORD and QFT results were not used for patient management or for classification of study participants as TB or ORD. At enrolment, all subjects had 3 ml of blood drawn for QFT. IFN- γ levels in QFT supernatants were measured using the QFT ELISA. As instructed by the manufacturer (Cellestis, Australia; now Qiagen, Germany), tests were regarded as positive for TB infection if the difference between the TB antigen stimulated and unstimulated (Nil) supernatant was greater than or equal to 0.35 IU/ml but greater than or equal to 25% of the Nil value. The tests were regarded as negative if that difference was <0.35 IU/ml and less than 25% of the Nil value, provided that the value of mitogen stimulated supernatant was ≥0.5 IU/ ml after subtraction of the unstimulated value as per the manufacturer’s manual [16]. The QFT analysis software, version 2.50 was used for analysis.

### Luminex immunoassay

The levels of 10 host markers were evaluated in serum and saliva samples for all study participants using a 10 plex customized kit from Bio-rad Laboratories (Hercules, CA, USA) on the Bio-Plex platform (Bio Rad). These included interferon gamma (IFN-γ), interleukin (IL) -2, 5, 6, granulocyte colony stimulating factor (G- CSF), granulocyte monocyte colony stimulating factor (GM-CSF), macrophage inflammatory protein (MIP)-1α and β, vascular endothelial growth factor (VEGF) and tumor necrosis factor alpha (TNF-α). As recommended by the manufacturer, the serum and saliva samples were diluted 1:4. Samples from one study participant were tested on the same plate. The Bio Plex Manager software, version 6.1 was used for bead acquisition and analysis of median fluorescent intensity.

### Statistical analysis

Data were analyzed using GraphPad Prism, version 6.01 (GraphPad Soſtware, California, USA) and the statistica software (StatSoft, USA). Differences in analyte levels between the TB patients and participants without TB disease or between the marker levels detected in saliva and serum levels were evaluated by the Mann-Whitney U test for nonparametric data analysis. The diagnostic accuracy of the markers was investigated by receiver operator characteristics (ROC) curve analysis. Optimal cut-off values, sensitivity and specificity were selected based on the highest likelihood ratio. The General discriminant analysis technique (GDA) was used to evaluate the accuracy of combinations between different biomarkers for the diagnosis of TB disease https://documents.software.dell.com/statistics/textbook/general-discriminant-analysis. This employed the training and test set approach whereby study participants were randomly split into a 70% training and 30% test set by the statistical software used in analysis (Statistica) https://documents.software.dell.com/statistics/textbook/general-discriminant-analysis. Differences between groups were considered significant if *p* values were <0.05.

## Results

### Study participants

Of the 78 participants, 39 (50%) had confirmed PTB by MGIT, 41 (53%) were females and 13 (16%) were HIV positive. Of all participants, 57 (73%) had a positive QFT as per the manufacturer’s recommended cut-off (≥0.35 IU/mL). The mean age of all participants was 32 ± 14 years. Eight of the 39 participants in the ORD group had abnormal chest X-rays which were consistent with active TB (Table 1). A comparison was done to evaluate if there was a difference in the markers produced by those participants who had No TB but had abnormal x-ray (*n* = 8) and normal x-ray and without TB (*n* = 31). The 8 participants with abnormal x-ray were believed to have other chest infections. There were no significant differences in the concentrations of the host markers between these individuals and other participants without TB disease and with normal chest x rays.

**Table 1:** Participant characteristics

Variable	All	PTB	No TB	
			LTBI	Uninfected
No. of participants	78	39	21	18
Female	41(53%)	17(22%)	11 (14%)	13 (17%)
HIV positive %	13 (17%)	5 (6%)	2 (3%)	6 (8%)
QFT positive	57 (73%)	36 (46%)	21 (27%)	0
Abnormal X-ray	42 (54%)	34 (44%)	7 (9%)	1 (1%)

Table 1: The distribution of study participants according to their sputum MGIT culture, Quantiferon and X-ray results is shown. All the TB patients were MGIT positive. No TB cases (all MGIT negative) were further classified as LTBI or uninfected individuals based on Quantiferon In Tube results. *LTBI* latent TB infection, *PTB* active pulmonary TB disease

### Expression of host markers in serum and saliva samples from all study participants

**Fig. 1:**
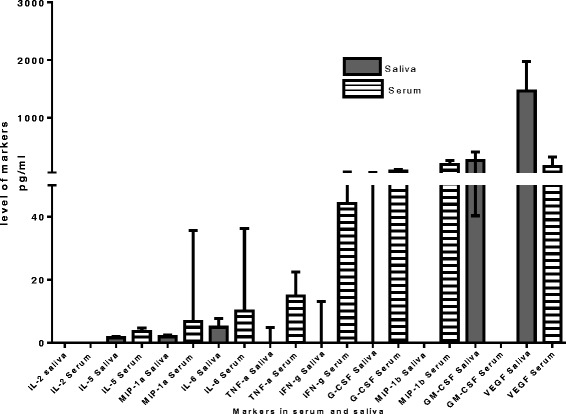
Median levels in pg/ml and interquartile ranges for all host markers in all study participants in both serum and saliva samples

When the concentrations of host markers detected in serum were compared to the levels detected in saliva in all study participants, significant differences were observed for all host markers, with *p* values ranging between <0.0001 and 0.03. Overall, the concentrations of IL-2 and IL-5 in all participants were low compared to other markers in both serum and saliva samples regardless of the participants’ TB status. However, the levels of IL-2 and IL-5 markers were comparably higher in serum. The concentrations of GM-CSF and VEGF were significantly higher in saliva in comparison to serum, whereas the concentrations of the other eight markers (IL-2, 5, 6, G-CSF, IFN-γ, MIP-1α, MIP-1β, TNF-α) were significantly higher in serum (Fig. 1, Table 2).

**Table 2:** Median levels of host markers were significantly different between saliva and serum samples from all participants, regardless of TB disease status

Marker	Median (pg/ml) Saliva	Median (pg/ml) serum	*p*-value
IL-2	0.0 (0.0–46.2)	0.0 (0.0–121.0)	0.03
IL-5	1.6 (0.0–25.0)	3.6 (0.6–27.5)	<0.0001
IL-6	4.9 (2.4–7.8)	10.17 (6.5–36.3)	<0.0001
G-CSF	0.0 (0.0–50.1)	72.5 (52.2–103.7)	<0.0001
GM-CSF	261.2 (40.4–409.1)	0.0 (0.0–0.0)	<0.0001
MIP-1a	1.9 (1.1–2.5)	6.7 (4.2–35.6)	<0.0001
MIP-1b	0.0 (0.0–0.0)	187.1 (114.4–260.4)	<0.0001
VEGF	1467.2 (678.4–1975)	154 (0.0–322.2)	<0.0001
TNF-a	0.0 (0.0–4.8)	14.8 (8.2–22.5)	<0.0001
IFNg	0.0 (0.0–13.1	44.3 (8.1–60.6)	0.0003

Table 2: Shown are the median marker levels (inter quartile ranges in parenthesis) for markers that were significantly different in serum compared to saliva in all participants, regardless of TB disease status

When the concentrations of the host markers detected in saliva were compared to the concentrations obtained in serum, but only in the TB patients, significant differences were observed for all markers (Table 3).

**Table 3:** Median levels of markers were significantly different between saliva and serum for all active pulmonary TB participants

Marker	Median (pg/ml) Saliva	Median (pg/ml) serum	*p*-value
IL-5	3.7 (2.7–4.7)	1.6 (0.6–2.7)	<0.0001
IL-6	5.1 (2.4–9.5)	33.1 (12.9–60.3)	<0.0001
G-CSF	0.0 (0.0–40.5)	76.1 (53.2–123.0)	<0.0001
GM-CSF	257.9 (77.9–428.9)	0.0 (0.0–0.0)	<0.0001
MIP-1a	1.9 (1.1–2.6)	6.41 (4.2–35.6)	<0.0001
MIP-1b	0.0 (0.0–0.0)	178.1 (98.7–245.8)	<0.0001
VEGF	1486 (104–1960)	224.6 (93.2–654.9)	<0.0001
TNF-a	0.0 (0.0–8.5)	19.6 (7.4--26.6)	<0.0001
IFNg	0.0 (0.0–15.6)	43.3 (0.94–74.3)	0.0003

Table 3: Shown are the marker levels that are significantly different (median levels and inter quartile ranges (in parenthesis) in serum compared to saliva in only active pulmonary TB participants with Area Under the ROC curve ranging between 0.73–0.97

**Fig. 2:**
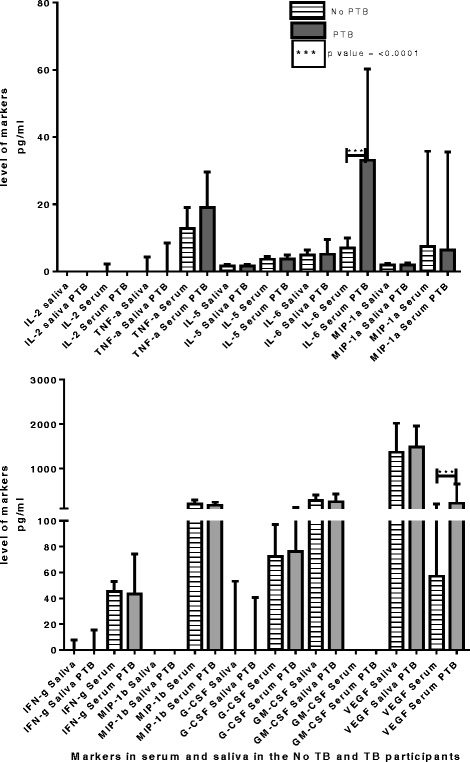
Median and inter-quartile ranges (pg/ml) of all 10 markers showing the differences between the active TB group (PTB) and the No PTB group (those with other respiratory diseases; ORD) in serum and saliva of all the study participants

When the values obtained in the two sample types were compared, but only in the no TB patients, significant differences were observed for all markers with AUCs greater than 0.73 (Table 4) with a *p*-value ranging between <0.0001 and 0.0003 (Fig. 2).

**Table 4:** Median levels of markers were significantly different between saliva and serum for all participants

Marker	Median (pg/ml) Saliva	Median (pg/ml) serum	*p*-value
IL-5	3.6 (2.7–4.7)	1.6 (0.6–2.1)	<0.0001
IL-6	4.8 (2.1–5.4)	6.9 (5.6–9.9)	<0.0001
G-CSF	0.0 (0.0–53.2)	72.5 (49.1–97.1)	<0.0001
GM-CSF	282.2 (36.3–405.5)	0.0 (0.0–0.0)	<0.0001
MIP-1a	1.9 (1.3–2.3)	7.39 (3.9–35.8)	<0.0001
MIP-1b	0.0 (0.0–0.0)	201.9 (127.5–297.3)	<0.0001
VEGF	1368 (550.5–2020)	57.1 (0–208.6)	<0.0001
TNF-a	0.0 (0.0–4.3)	12.7 (8.5–19.1)	<0.0001
IFNg	0.0 (0.0–7.7)	15.3 (10.4–52.9)	0.0003

Table 4: Shown are the marker levels that are significantly different (median levels and ranges (in parenthesis) in serum compared to saliva in only participants with other respiratory diseases with Area Under the ROC curve ranging between 0.74–1

### Abilities of host markers detected in serum and saliva in the diagnosis of TB disease

When the concentrations of host markers detected in serum samples were compared between the TB patients and individuals with ORD with the Mann Whitney U test, the concentrations of IL-6 and VEGF were significantly different between the two groups. The concentrations of IL-6 were significantly higher in active PTB group (*p* = <0.0001) whereas the concentrations of VEGF were significantly higher in ORD group (*p* = 0.001). When the accuracy of the serum biomarkers were assessed by ROC curve analysis, the potentially most useful individual host markers, as determined by area under the ROC curve (AUC >0.70) were IL-6 and VEGF (Table 5).

**Table 5:** Median levels of the most promising host markers detected in serum samples from patients with active TB group (TB) or ORD group and accuracies in the diagnosis of TB disease

Groups	TB Vs ORD	
Marker	IL-6 Serum	VEGF Serum
ORD (median pg/ml)	6.9 (5.6–9.9)	57.06 (0–208.6)
PTB (Median pg/ml)	33.11 (12.9–60.2)	224.6 (93.2–654.9)
*p*-value	<0.0001	0.001
AUC	0.85	0.71
Cut off	>36.4	>573.8
Sensitivity	46.15	28.21
Specificity	97.44	97.44

Table 5: Shown are the median and ranges (in parenthesis) levels of serum markers that were significantly different between the TB patients and the group with other respiratory diseases (ORD) and area under the curve (AUC) showing the diagnostic performance of the markers

When the concentrations of host markers detected in saliva samples were compared between the active TB group and ORD group, no significant differences were observed for all the 10 markers investigated (Table 6).

**Table 6:** Median marker levels in saliva between the active PTB group and those with other respiratory infections and area under the curve (AUC) showing diagnostic performance of markers

Marker	Median (pg/ml) PTB	Median (pg/ml) ORD	*p*-value	AUC	Sensitivity	Specificity	Cut off
IL-2	0.0 (0.0–0.0)	0.0 (0.0–0.0)	0.57	0.53	89.74	15.38	<0.38
IL-5	1.6 (0.6–2.1)	1.6 (0.6–2.1)	0.52	0.54	12.82	97.44	<0.12
IL-6	5.11 (2.4–9.5)	4.8 (2.1–6.4)	0.23	0.58	25.64	89.74	<2.1
G-CSF	0.0 (0.0–40.5)	0.0 (0.0–53.2)	0.64	0.53	17.95	92.31	>86.61
GM-CSF	257.9 (77.9–428.9)	282.2 (36.3–405.5)	0.65	0.53	20.51	92.31	<6.53
MIP-1a	1.9 (1.1–2.6)	1.9 (1.3–2.3)	0.69	0.53	7.69	97.44	<0.55
MIP-1b	0.0 (0.0–0.0)	0.0 (0.0–0.0)	0.61	0.51	97.44	7.69	<31.06
VEGF	1486 (1004–1960)	1368 (550.5–2020)	0.24	0.58	28.21	92.31	<571.3
TNF-a	0.0 (0.0–8.5)	0.0 (0.0–4.3)	0.45	0.54	84.62	33.33	<5.32
IFNg	0.0 (0.0–15.6)	0.0 (0.0–7.7	0.18	0.57	71.79	41.03	<0.47

Table 4: Shown are the saliva marker levels (median levels and inter quartile ranges (in parenthesis) comparing active TB (PTB) participants with other respiratory diseases (ORD) with Area Under the ROC curve ranging between 0.51–0.58

### Differences in the expression of host biomarkers in individuals with TB disease, LTBI and no *Mtb* infection

**Fig. 3:**
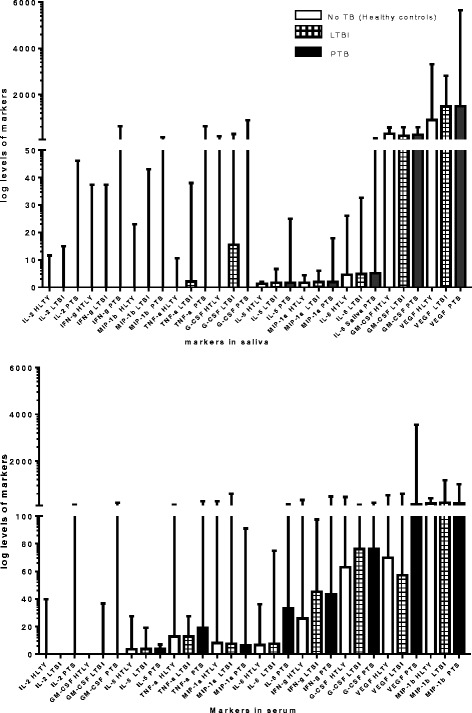
Median and interquartile ranges of all 10 markers showing the differences between the active TB group (PTB), LTBI and the uninfected (No TB) group in serum or saliva of all the study participants

Of the 39 participants in the No TB group, 18 had a negative QFT result. We compared the concentrations of host markers detected in serum and saliva in individuals with active PTB, LTBI or no *Mtb* infection, considering the QFT positive non-TB patients as LTBI. When the concentrations of host markers detected in saliva were compared between any two groups (PTB vs LTBI or PTB vs No *Mtb* or LTBI vs No *Mtb*) none of the host markers investigated (with the exception of IFN-γ showed significant differences between the two groups (Table 7, Fig. 3).

**Table 7:** Median markers levels in serum and saliva that were significantly different between the active TB group (TB), latent TB infection and uninfected (No TB infection) group

Groups	PTB vs LTBI		PTB vs HE	LTBI vs HE
Marker	IL-6 Serum	VEGF Serum	IFN-γ saliva	
PTB Median pg/ml	33.11 (12.9–60.3)	224.6 (93.2–654.9)	0 (0.0–15.6)	
LTBI (median pg/ml)	7.6 (5.6–10.7)	57.1 (0.0–230.3)		0 (0–16.7)
Uninfected (median pg/ml)	6.7 (4.3–8.7)	69.8 (0.0–198.9)	0.0 (0.0–0.0)	
*p*-value	<0.0001	0.001	0.03	0.04
AUC	0.85	0.71	0.65	0.66
Cut off	>36.4	>573.8	<0.47	<0.47
Sensitivity	46.15	28.21	88.9	88.9
Specificity	97.44	97.44	41	43

Table 6: Shown are the median levels of IL-6, VEGF IFN- γ and inter quartile ranges (in parenthesis) for serum markers that were significantly different between the active PTB, latent tuberculosis infection (LTBI) and the HE (No TB) group. Area under the curve (AUC) showing the diagnostic performance of markers

**Fig. 4:**
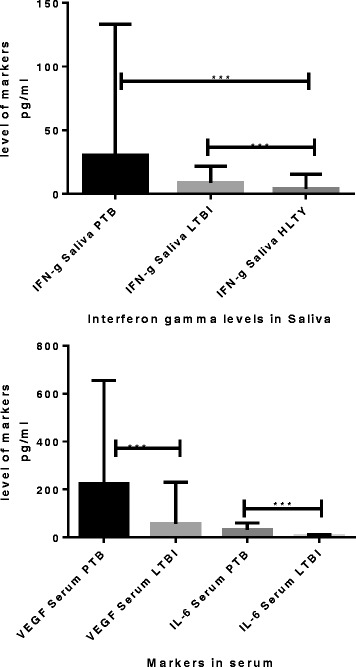
Top: Levels of markers significantly different in the serum samples of pulmonary TB cases and latent TB disease, and below: Level of markers that were significantly different the different TB disease in saliva

**Fig. 5:**
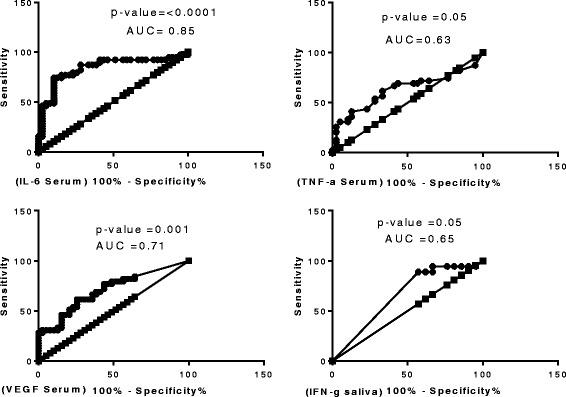
ROC curves with *p* values and Area Under the Curve (AUC) values for those markers that were significantly different in active TB disease and No TB disease

When the concentrations of host markers detected in serum were compared between any two groups (PTB vs LTBI or PTB vs No *Mtb* or LTBI vs No *Mtb*) the concentrations of IL-6 and VEGF showed significant difference between the two groups (Table 7). Except for IFN-γ (*p* value 0.04) in saliva (Table 7, Fig. 4), serum levels of IL-6 and VEGF were significantly different between the active TB patients and latent TB infection or uninfected (No TB individuals) with *p*-values ranging between <0.0001 to 0.01 and area under the ROC curve ranging between 071 and 0.86 (Table 7, Figs. 4, 5).

### Utility of marker combinations for diagnosis of TB disease

When the general discriminant analysis technique was used to assess the predictive abilities of combinations of serum and salivary biomarkers for TB disease, a 5-marker biosignature comprising of serum IL-6, MIP-1β, VEGF and saliva G-CSF and MIP-1α, diagnosed TB disease in the training sample set (*n* = 54; *n* = 27 TB cases and *n* = 27 ORD), with a sensitivity of 81.5% (22/27) and specificity of 100% (27/27), and with a sensitivity of 50% (6/12) and specificity of 75% (9/12) in the test sample set (*n* = 24, *n* = 12 TB cases and *n* = 12 ORD).

When only the saliva biomarkers were taken into consideration, a 3-marker model comprising of salivary G-CSF, TNF-α and VEGF diagnosed TB disease in the training sample set (*n* = 54; *n* = 27 TB and *n* = 27 ORD) with a sensitivity of 63% (17/27) and a specificity of 63% (17/27). In the test sample set (*n* = 24; *n* = 12 TB, *n* = 12 ORD) however, the sensitivity and specificity of the 3-marker salivary biomarker model were only 42% (5/12) and 75% (9/12) respectively.

**Fig. 6:**
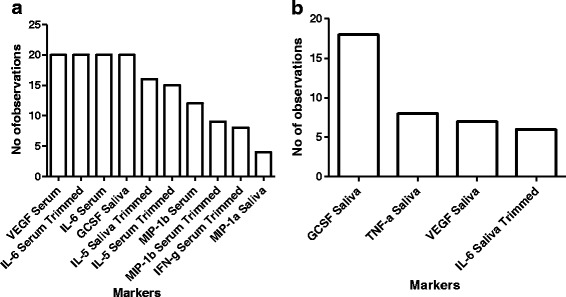
(**a**) Bar graph showing frequency of analytes in the GDA models generated from combining saliva and serum samples, (**b**) frequency of analytes in models generated when the data obtained from the host markers detected in saliva only

The most frequently occurring markers in the TB disease predictive combinations comprised of serum IL-6, VEGF, and MIP-1β as well as salivary G-CSF and MIP-1α (Fig. 6).

## Discussion

The search for biomarkers of TB disease and infection requires the investigation and research on non-sputum samples for TB diagnosis [17]. Early in biomarker research, IFN-γ took the lead as a relevant TB biomarker. It has however shown not to be a reliable biomarker if not used in conjunction with other markers. With the search for other biomarkers in TB research on the lead, there’s a need for a non-sputum biological sample and biomarker [18]. This study evaluated whether saliva could serve as an alternative specimen to serum for TB diagnosis. This owes to its non invasive nature of collection which can be applicable in children and the very ill groups [19]. Recently, studies evaluating the relevance of salivary host markers have shown promising markers that could be incorporated in a point-of-care test following validation [10, 11] and [13]. Overall, the levels of markers were higher in serum for all participants except for GM-CSF and VEGF. This confirms the findings by Phalane et al. [13]. This study found increased levels of VEGF and IL-6 in serum samples of active PTB patients compared to those with other respiratory diseases. These were also identified in previous studies [10, 11] and [13]. Increased VEGF in serum of patients with active TB makes it a useful prognostic indicator in granulomatous diseases, such as TB as identified by [20]. This suggests its role in pathogenesis of pulmonary TB in the development of the chronic inflammatory reaction.

In line with a study by Phalane et al. [13], increased saliva levels of GM-CSF in this study are relevant to enhance T cell responses, regulate phagocytosis and innate immune responses in alveolar macrophages [21]. High GM-CSF levels have also been identified in saliva of patients with oral squamous cell carcinoma [22].

Serum levels of MIP-1α, IL-6, TNF-α, IFN-γ and G-CSF were noticeably higher than were in saliva. IL-6 and TNF-α are pro-inflammatory cytokines which have a role in granuloma formation [23]. MIP-1α and β are important chemo attractants for lymphocytes to the site of TB infection leading to suppression of *Mycobacterium* growth [24]. Findings by other investigators [25, 26] also identified MIP-1α and β to be produced in high amounts in TB disease. IFN-γ on the other hand is important in promoting antigen presentation and recruiting T helper and cytotoxic T cells involved in killing the bacilli. In combination with TNF-α, they activate macrophages in order to kill intracellular pathogens and induce production of reactive nitrogen intermediates [27]. Serum levels of markers are insufficient to base on for *Mtb* complex diagnosis as we can’t pin their origin entirely to one immune condition such as PTB [28]. For this reason, salivary markers are being evaluated for their use in PTB disease diagnosis.

When salivary marker levels were evaluated for their ability to differentiate between active TB disease from No TB disease, there was no significant difference between the individual marker levels. Comparing with serum, the concentrations of IL-6 and VEGF were significantly different between the active TB disease group and the ORD group. This may suggest that salivary biomarkers may not be very useful individually in the diagnosis of TB disease. This may however, only apply to the markers investigated in the current study as previous reports identified salivary biomarkers which showed potential in the diagnosis of TB disease (Phalane et al., [13] Jacobs et al., [10] Jacobs et al. [11]). When biomarkers were used in combinations however, a biosignatures containing salivary biomarkers and combinations between saliva and serum markers showed potential. This correlates with findings by [29] and [30] which reported high VEGF levels in active TB. Our findings also suggest that Interleukin-6 as noted by previous studies by Jacobs et al., [10] and Phalane et al.*,* [13] may be relevant for TB diagnosis. IL-6 appeared in predictive combinations and is known to stimulate the secretion of IFN-γ, a crucial cytokine in the activation of macrophages infected with *M.tb* [31]. This correlates with findings by [24] who identified increased serum levels of IL-6 in active TB patients. IL-2 on the other hand was below detectable levels for both serum and saliva. It is a relevant T cell growth factor, macrophage activator [23] and contributes to T cell memory. Previous studies found IL-2 production to be reduced among active PTB patients [32]. This may explain the low IL-2 levels shown given that all samples used were baseline from TB suspects.

More investigations are required including studies done on biomarkers other than the ones investigated in the current study, given the potential that a diagnostic tool based on saliva may contribute to the management of TB disease in all patient types especially those with difficulty in providing sputum samples such as children. Such future studies should focus on the identification of biosignatures, rather than individual biomarkers given that these inflammatory biomarkers may not be highly specific for TB, especially when used alone. Validated salivary biosignatures could then be incorporated into point-of-care tests for the diagnosis of TB disease (Sutherland et al. [33]).

## Conclusion

In conclusion, saliva may be an important alternative diagnostic sample for PTB diagnosis and biomarker discovery. Our findings suggest that biosignatures comprising of combinations between different host markers detected in saliva may be useful for the diagnosis of PTB disease. We confirmed that there are highly significant differences in the concentrations of some biomarkers expressed in saliva in comparison to serum levels and therefore biomarkers that do not show potential in serum samples may not necessarily yield the same results in saliva. Therefore saliva warrants further exploration as a sample for biomarker TB biomarker discovery given that it is a very easily accessible sample and can be collected from all patient types, and immunological assays based on saliva may be easily translated into simple, culture-free point-of-care tests. Furthermore, saliva may be a more informative sample type than serum as it is a mucosal/airway linked sample and therefore is closer to the site of disease than peripheral blood samples. Our findings require further investigation in future larger prospective studies.

## Abbreviations

AUC: Area under the ROC curve
*Mtb*: *Mycobacterium tuberculosis*
ORD: Other respiratory diseases
PTB: Pulmonary tuberculosis
